# Development of Anti-Virulence Therapeutics against Mono-ADP-Ribosyltransferase Toxins

**DOI:** 10.3390/toxins13010016

**Published:** 2020-12-25

**Authors:** Miguel R. Lugo, Allan R. Merrill

**Affiliations:** Department of Molecular and Cellular Biology, University of Guelph, Guelph, ON N1G 2W1, Canada; mlugoa@gmail.com

**Keywords:** mono-ADP-ribosyltransferase toxins, anti-virulence agents, bacterial toxins, protein crystallography, virtual screening, drug discovery

## Abstract

Mono-ADP-ribosyltransferase toxins are often key virulence factors produced by pathogenic bacteria as tools to compromise the target host cell. These toxins are enzymes that use host cellular NAD^+^ as the substrate to modify a critical macromolecule target in the host cell machinery. This post-translational modification of the target macromolecule (usually protein or DNA) acts like a switch to turn the target activity on or off resulting in impairment of a critical process or pathway in the host. One approach to stymie bacterial pathogens is to curtail the toxic action of these factors by designing small molecules that bind tightly to the enzyme active site and prevent catalytic function. The inactivation of these toxins/enzymes is targeted for the site of action within the host cell and small molecule therapeutics can function as anti-virulence agents by disarming the pathogen. This represents an alternative strategy to antibiotic therapy with the potential as a paradigm shift that may circumvent multi-drug resistance in the offending microbe. In this review, work that has been accomplished during the past two decades on this approach to develop anti-virulence compounds against mono-ADP-ribosyltransferase toxins will be discussed.

## 1. Introduction

The current situation promises significant difficulty with bacterial resistance to the current antibiotic libraries and a drug industry that cannot provide new compounds, especially considering the emergence of multi-drug-resistant bacteria [1,2,3]. In recent years, the burgeoning bacterial drug resistance problem has disrupted the pharmaceutical pipeline in the pursuit of novel antibacterials [4,5,6,7]. The current reservoir for developing novel anti-infective agents consists mostly of antibiotic derivatives in the various classes including *β*-lactams, quinolones, macrolides, and glycopeptides (3) [8,9]. The U.S. has witnessed an epidemic of methicillin-resistant *Staphylococcus aureus* (MRSA) infections [10,11], and these are spreading through community centers, schools, gymnasiums, and clinics, within a matter of weeks. The elevated incidence of flesh-eating diseases [12], fatalities due to contaminated drinking water [13], *Listeria monocytogenes* contamination of foods [14], *Escherichia coli* O157 contamination of meats (500 deaths annually in the U.S.) [15], *Salmonella*-related food poisoning such as vegetable contamination (4 million U.S. cases/y) [16], and the appearance of multi-drug-resistant bacteria [17] clearly necessitate immediate action to search for novel antimicrobials. In hospitals, *Pseudomonas aeruginosa* is a notorious, opportunistic, multi-drug-resistant human pathogen frequently encountered in nosocomial infections; the third most common bacterial isolate from blood-borne infections; and the most frequent cause of nosocomial pneumonia [18,19,20]. It is known to form drug-immune biofilms and to cause urinary tract infections; ear, nose, and throat infections; and cardiovascular and bloodstream infections. It devastates immune-compromised individuals such as those suffering from cystic fibrosis, cancer, burns, and major surgery [17,21,22,23].

Many bacterial pathogens cause infection using toxins as tools to modify host cells. The bacterial mono-ADP-ribosyltransferases (mARTs) are a protein exotoxin family that covalently modify host proteins, DNA, RNA, or even antibiotics [24,25,26]. Each mART toxin produces a unique pathology by modification of one or more specific host proteins [27]. We developed a strategy founded on protein-fold-recognition methods that identifies prospective, new mART toxin family members through data mining bacterial genomes [28,29]. These data-mined toxins serve as targets of novel therapeutics for disease treatment and can shed light on the toxin–enzyme active-site architecture and the mechanisms of host cell invasion and cytotoxic activity [28,29,30,31,32,33,34,35,36,37,38].

**Figure 1:**
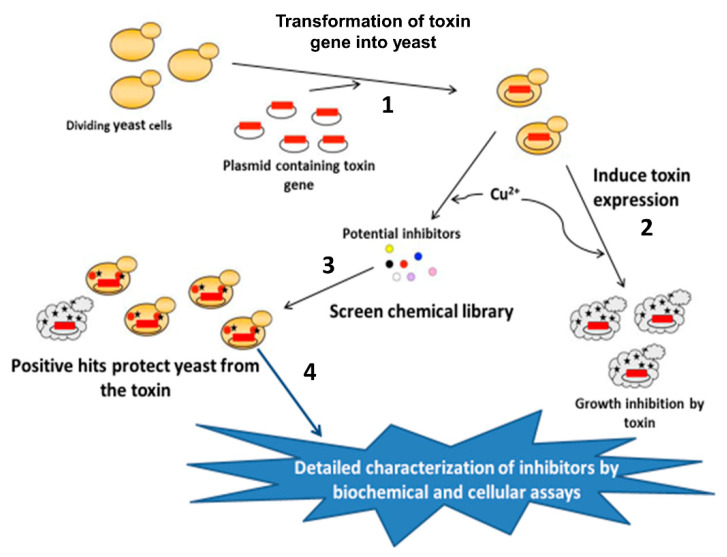
Summary of the yeast synthetic-lethal phenotypic screening assay to characterize and identify new bacterial virulence factors and small-molecule inhibitors against mono-ADP-ribosyltransferase (mART) toxins. (1) *S. cerevisiae* is grown to log phase and transformed with linear CUPI plasmid and toxin gene insert flanked by 20 base pairs homologous to the plasmid. (2) Successful transformants are grown in selective media where yeast culture is supplemented with Cu^2+^ to induce a growth-defect phenotype. (3) Alternatively, small-molecule compounds are added to the culture medium upon induction of the toxin gene to reverse the phenotype. (4) Inhibitors shown to reverse the growth-defect phenotype in yeast are later characterized in target host cell lines to define their therapeutic potential.

To facilitate mART toxin discovery and characterization as targets for anti-virulence therapy, *Saccharomyces cerevisiae* (*S. cerevisiae*) was developed as a platform for toxin gene expression controlled by the Cu^2+^-inducible *CUP1* promoter, where an active toxin in yeast causes a growth-defect phenotype (Figure 1) [39,40]. This system is a tool to confirm the cytotoxicity of data-mined, putative mART toxins and to screen for anti-virulence compounds in the yeast model. The yeast cytotoxicity data are then compared with toxin activity against the target cell lines to provide a correlation from model (yeast) to relevant cell system [31,32,37,40]. Importantly, the best compounds have proven to function as strong inhibitors of both the diphtheria toxin (DT) and the cholera toxin (CT) groups. However, there are fundamental differences between the DT- and CT-group active-site architectures that necessitate a specific inhibitor design for each group. Furthermore, the variability in the CT-group members also requires a specific inhibitor design for each subgroup [24,31,32,37].

## 2. Anti-Virulence Approach to Inhibitor Development

**Figure 2:**
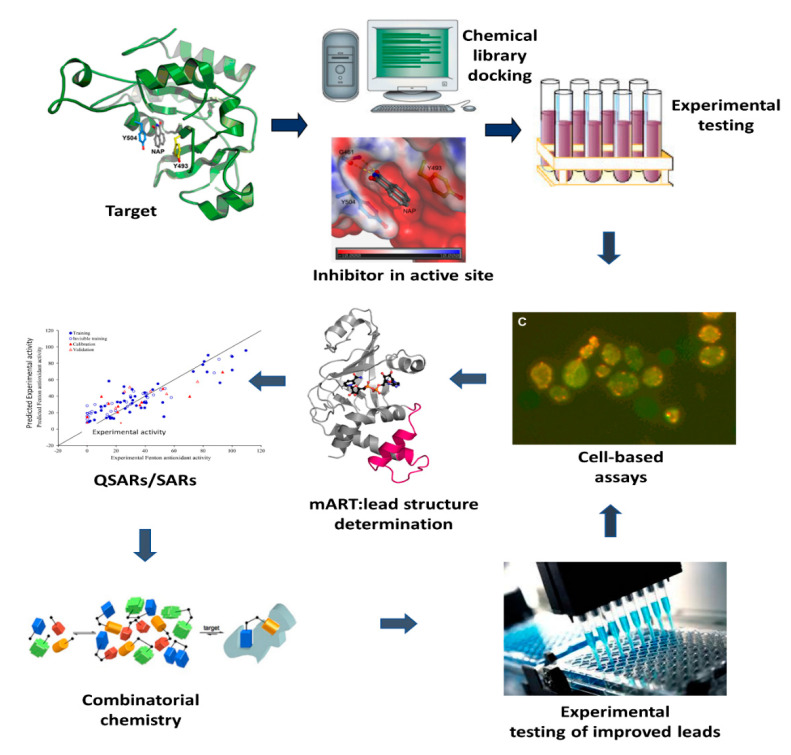
mART toxin anti-virulence development. mART inhibitors are gleaned from virtual screening by targeting high-resolution structure of a toxin-inhibitor complex and directed libraries, followed by in vitro testing against mART targets. Promising compounds are tested for protection of the target host cells from mART cytotoxicity and for chemical toxicity. Leads are co-crystallized with their cognate toxin followed by lead optimization through combinatorial chemistry and retesting. Periodically, the best leads are also tested for efficacy and toxicity in animal infection models and are then made available for pharmaceutical partners. QSARs/SARs refers to Quantitative Structure-Activity Relationships/Structure-Activity Relationships.

A novel approach with the potential for a paradigm shift to treating bacterial diseases (Figure 2) is to target mART toxins using anti-virulence strategies [41,42]. Anti-virulence compounds promise significant advantages over conventional antibiotics [43]. Firstly, these inhibitors target specific mechanisms in the target pathogen that cause infection rather than inactivating an essential metabolic pathway [44]. Disarming microorganisms of their virulence factors without threatening their survival alleviates selection pressure, reducing the frequency for drug-resistant mutations [45]. Secondly, virulence-specific therapeutics cause less harm to host microbiota than conventional antibiotics. Thus, the anti-virulence approach features a paradigm shift in the management and treatment of bacterial infections and diseases. This unorthodox approach makes pathogenic bacteria irrelevant by disarming their weaponry, providing additional time for the host’s immune system to clear the pathogen. To date, we have forged a targeted inhibitor library against a contingent of mART toxins; the best compounds provide a protective shield for target cells from toxin-induced death [39,46]. These findings provide proof-of-principle that bacterial toxin inhibitors may function to reduce the virulence of bacterial pathogens.

## 3. Inhibitors against mART Toxins

### 3.1. Pseudomonas aeruginosa Exotoxin A (ExoA)

**Figure 3:**
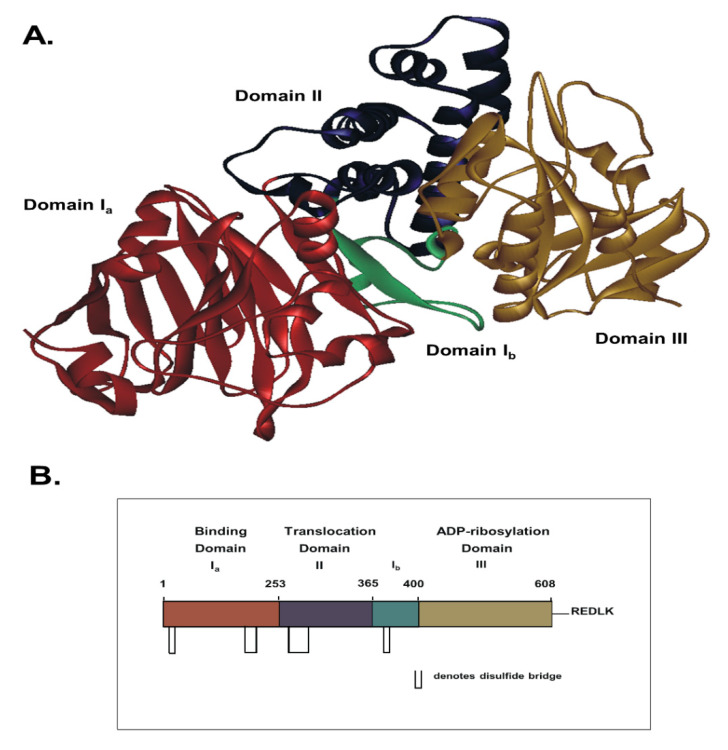
X-ray and domain structure of *P. aeruginosa* exotoxin A (ExoA). (**A**) X-ray structure of full-length, mature ExoA at 1.6 Å (PDB:1IKQ) showing the four domains. (**B**) Primary structure cartoon showing the relationship between structural and functional domains of ExoA.

**Figure 4:**
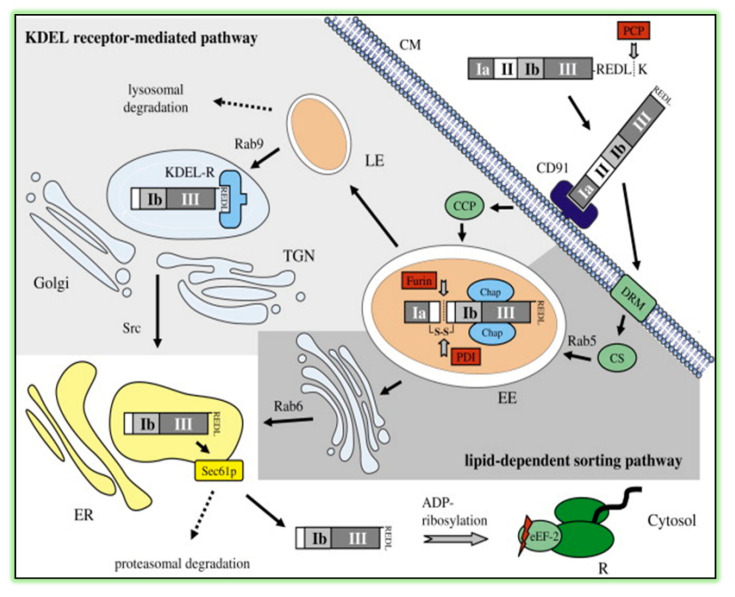
Cytotoxic pathways of *Pseudomonas* exotoxin A (ExoA). After cleavage of the C-terminal lysine (K) by plasma carboxypeptidases (PCPs), ExoA binds to the CD91 receptor on the cell membrane (CM) and can then exploit different pathways to reach the endoplasmic reticulum (ER). On the one side, ExoA is internalized via clathrin-coated pits (CCPs) into the cell. This is followed by furin cleavage in the early endosomes (EEs) in cooperation with protein disulfide isomerase (PDI) and chaperones (Chaps). Then, the enzymatic active ExoA fragment travels via late endosomes (LEs) in a Rab9-dependent manner to the trans-Golgi network (TGN). After binding to the KDEL receptor (KDEL-R), ExoA is transported to the ER under control of the tyrosine kinase Src. On the other side, CD91-bound ExoA can associate with detergent-resistant microdomains (DRMs) and is transported via caveosomes (CSs) to the EE in a Rab5-dependent manner. After cleavage in the EE, the toxic ExoA fragment directly travels to the ER via a lipid-dependent sorting pathway under the control of Rab6. ExoA fragments in the ER are secreted via the translocon Sec61p into the cytosol, where they inhibit the protein synthesis by ADP-ribosylating the eukaryotic elongation factor 2 (eEF2) at the ribosomes (Rs). This finally leads to apoptosis of the host cell. Figure was taken from reference [60].

ExoA is an important virulence factor produced by *Pseudomonas aeruginosa* that possesses an LD_50_ of 0.2 μg/kg upon intraperitoneal injection into mice [47]. ExoA inhibits protein synthesis [48], to cause a host hyper-immune response [49], and to induce Bak-dependent apoptosis in host cells [50]. The holotoxin is composed of three domains: receptor-binding (Domain I), membrane translocation (Domain II), and enzyme activity (Domain III; Figure 3). It is produced and secreted as a catalytically inactive 638-residue proenzyme [51] and is activated by host plasma carboxypeptidases by cleavage of a terminal Lys [52]. Activated toxin then binds to CD91 (also known as α2MR/LRP receptor), is internalized into clathrin-coated pits, and is transported to early endosomes associated with Rab5 [53] (Figure 4). Upon endosome acidification, ExoA dissociates from CD91, is cleaved at Arg279, and is reduced (critical disulfide bond), thus, producing a catalytically active 37-kDa fragment. This fragment travels the KDEL retrograde pathway and a Rab9-dependent route to the trans-Golgi network and to the endoplasmic reticulum (ER). The 37-kDa fragment finally reaches the host cell cytoplasm via the Sec61p translocon [54] and catalyzes the ADP-ribosylation of its target, eukaryotic elongation factor 2 (eEF2) [55] resulting in protein synthesis inhibition, cell cycle arrest [56], and host cell apoptosis [51,57]. X-ray structural and EM analyses revealed that ExoA mimics the normal interaction between eEF2 and the 80s ribosome [58,59].

A series of small, nonpolar compounds were developed as inhibitors of the ADP-ribosyltransferase activity of ExoA. The IC_50_ values for the compounds tested ranged from 87 nM to 484 μM for 1,8-naphthalimide (NAP) and CMP12, respectively [61]. NAP was a competitive inhibitor of the ADPRT reaction for the NAD^+^ substrate with a K_i_ of 45 ± 5 nM (K_D_ = 56 ± 6 nM). Furthermore, NAP was shown to bind within the ExoA active site by exhaustive dialysis, nuclear magnetic resonance (NMR), and electrospray mass spectrometry. Finally, a computer molecular model was generated with NAP bound within the ExoA active site based on the X-ray structure of ExoA with bound NAD^+^ substrate [61].

**Figure 5:**
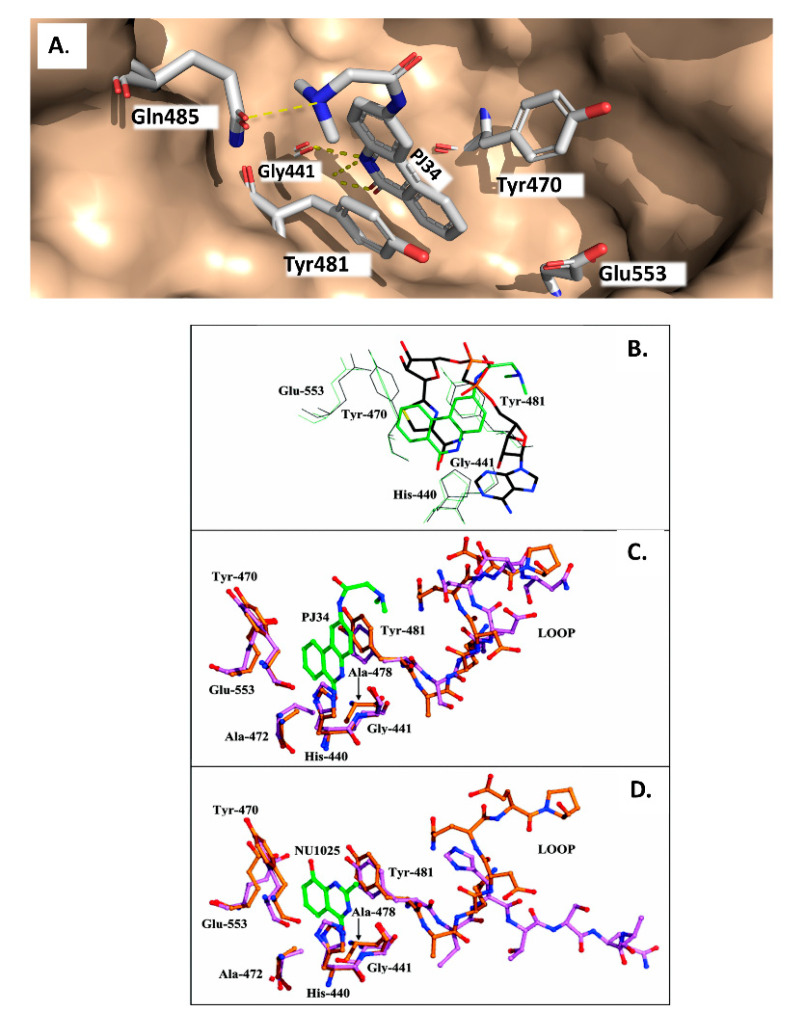
ExoA with PJ34 inhibitor. (**A**) PJ34 inhibitor binds into the nicotinamide pocket and is held in place through hydrophobic contacts and H-bonds including two notable H-bonds with Gly441 (main chain) and Gln485 (side-chain oxygen) (PDB:1XK9). The Tyr481 phenyl side-chain shows van der Waals interactions with PJ34 (4 Å away) and the two aromatic rings exhibit π–π associations. Tyr470 is more distant from PJ34 at a 40° angle and does not provide much stability to the bound inhibitor. (**B**) Superposed structure of PE24H-PJ34 (shown in lime green) on the ExoA Domain III in complex with β-TAD, an NAD^+^-analogue (shown in black, PDB entry 1AER (8)). β-TAD mimics nicotinamide of NAD^+^ with its thiazole substituent. (**C**,**D**) Superposed structure of PJ34-PE24H (shown in orange) with (**C**) DT (shown in pink, PDB entry 1TOX (13)) or (D) PARP-NU1025 (shown in pink, PDB entry 4PAX). A catalytic loop of ExoA (termed LOOP) is shown and includes residues 482 to 487, 66 to 71 in DT or 908 to 913 in PARP. In the DT comparison (**C**), the PJ34 inhibitor (green) is shown; for PARP, (**D**) the inhibitor NU1025, 8-hydroxy-2-methyl-3-hydro-quinazolin-4-one (green), is shown and the PJ34 ligand was omitted to demonstrate the similar orientation of a PARP inhibitor within the toxin active site as PJ34.

In a later study, Yates et al. (2005) characterized a small library of water-soluble compounds that mimic NAD^+^ substrate (nicotinamide ring) [62]. The best inhibitors featured an amide moiety embedded into a hetero-ring structure in a planar configuration (Table 1). Compounds that showed weaker inhibition against ExoA exhibited ring structures that were more flexible and less planar. The most potent inhibitor was PJ34 (N-[6-oxo-5,6-dihydro-phenanthridin-2-yl]-N,N dimethyl acetamide), a water-soluble phenanthridinone derivative with a 140 nM K_i_ value [62], and the structure of PJ34 bound to the toxin active site is shown in Figure 5. PJ34 binds into the nicotinamide pocket and is held in place through hydrophobic contacts along with two notable H-bonds with Gly441 (main chain) and Gln485 (side-chain oxygen) (Figure 5A). The phenyl of Tyr481 shows van der Waals interactions with PJ34 (4 Å away) and the two aromatic rings exhibit π–π associations. Tyr470 is more distant from PJ34 at a 40° angle and does not provide much stability to the bound inhibitor. Superposition of ExoA-PJ34 active-site structure with ExoA-βTAD^+^ structure clearly shows that the PJ34 inhibitor is mimicking the NAD^+^ substrate in the binding pocket of the enzyme (Figure 5B). Upon superposition of the ExoA catalytic domain with those of DT and PARP revealed that all enzymes share common NAD^+^ binding residues and catalytic residues as expected (Figure 5C,D); however, there is a notable difference between PARP and ExoA in the region of the catalytic loop of ExoA (Figure 5D). This loop is not shared with PARP and this may account for the target substrate differences between PARPs and the DT-class of mART toxins. This is a potential site for specific inhibitor development for these two enzyme groups.

**Figure 6:**
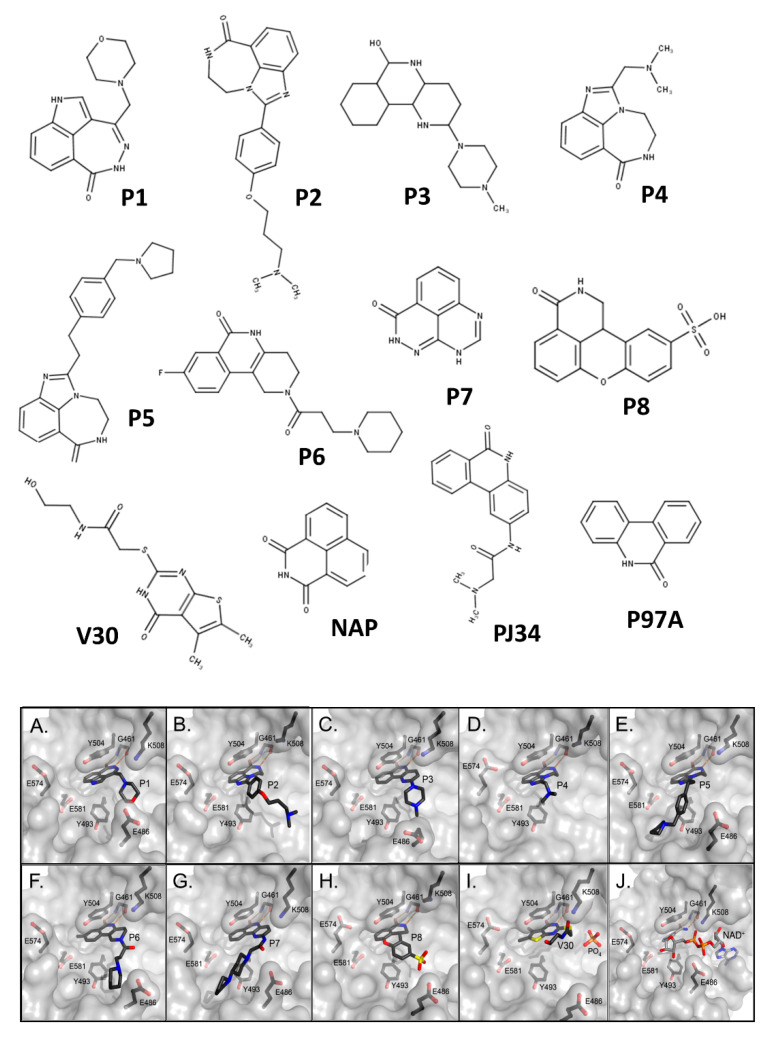
Chemical and X-ray structures of ExoA and cholix inhibitors. **Top panel:** The P-series (P1-P8) compounds are shown along with V30, the most active V-series compounds. Additionally, shown are two previously characterized ExoA inhibitors, 1,8-naphthalimide (NAP) and N-(6-oxo-5,6-dihydrophenanthridin-2-yl)-(N,N-dimethylamino) acetamide hydrochloride (PJ34). The chemical structures of a NAP derivative, 4-amino-NAP, and the parent compound for PJ34 (PJ97A) are also shown. **Bottom panel:** X-ray crystal structures of the catalytic fragment of cholix bound to inhibitor compounds. (**A**) cholix-P1, (**B**) cholix-P2, **(C**) cholix-P3, (**D**) cholix-P4, (**E**) cholix-P5, (**F**) cholix-P6, (**G**) cholix-P7, (**H**) cholix-P8, (**I**) cholix-V30, and (**J**) model of cholix-NAD^+^ complex. The inhibitors and the NAD^+^ substrate are shown with standard atom colors and nearby residues are shown as black sticks. Hydrogen bonds are shown in orange dashed lines. The model of the cholix-NAD^+^ complex is based upon the ExoA-NAD^+^ complex (PDB:3B78).

A thorough investigation of mART toxin inhibitors was subsequently completed for ExoA. In that study, there were two compound libraries that were used including the P-series, a small PARP inhibitor collection, and the V-series, a library generated from a virtual screen using cholix toxin with PJ34 inhibitor (PDB:2Q6M) as the receptor [39]. Additionally, two previous mART inhibitors, NAP and PJ34, were included in the study (Figure 6, top panel) along with 4-amino-NAP, a polar derivative of NAP, and PJ97A, the nonpolar parent of PJ34. Several compounds showed low nM K_i_ values (< 100 nM) against the NAD^+^ substrate for ExoA activity including NAP, P1, PJ34, P2, P6, and P3 (Table 2). The ability of these compounds to protect C38 human lung cells from ExoA was also assessed and P1, P6, NAP, V30, P4, and P5 all showed efficacy with EC_50_ values ranging from 2.9–16.7 μM. In summary, 8 compounds showed full protection of target cells against high doses of toxin, 6 compounds showed modest protection, and 11 compounds exhibited weaker protection [39].

**Figure 7:**
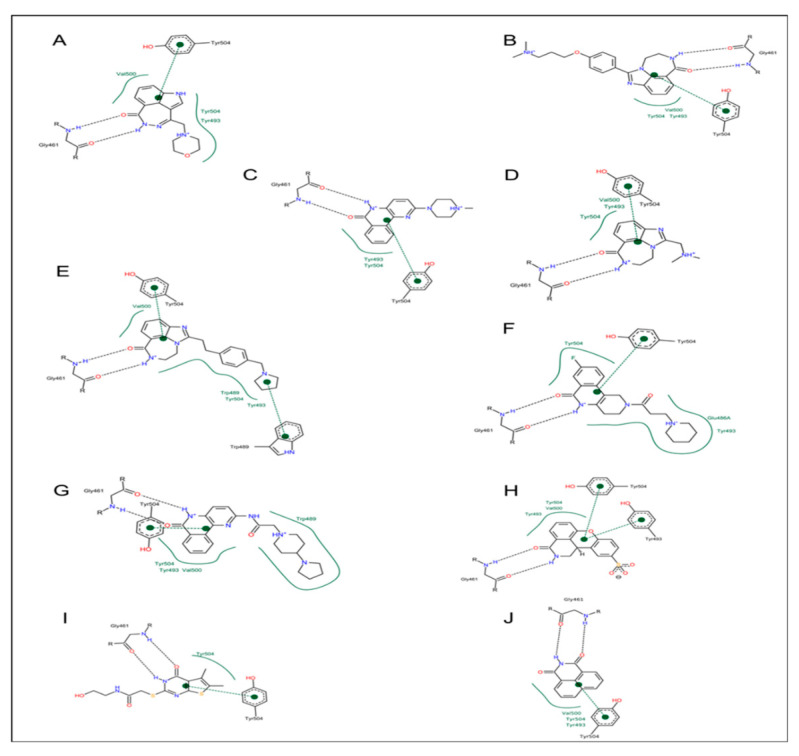
Two-dimensional chemical drawings of catalytic fragments of cholix-inhibitor complexes based on the corresponding crystal structures. (**A**) cholix-P1, (**B**) cholix-P2, (**C**) cholix-P3, (**D**) cholix-P4, (**E**) cholix-P5, (**F**) cholix-P6, (**G**) cholix-P7, (**H**) cholix-P8, (**I**) cholix-V30, and (**J**) cholix-NAP. Two-dimensional cholix-inhibitor visualization was achieved from the respective PDB files, using Open Babel (http://openbabel.sourceforge.net/) to convert these coordinates to structure data format and then drawing the complex using PoseView (http://poseview.zbh.uni-hamburg.de/).

High-resolution structures of compounds P1, P2, P3, P4, P5, P6, P7, and V30 were solved and provided validation of both the in vitro and cell-based inhibition data (Figure 6, bottom panel). These structures revealed that all compounds are competitive inhibitors of the NAD^+^ substrate and they dock/embed within the nicotinamide pocket of ExoA (cholix toxin is the crystallography model for ExoA). The important aspects of these inhibitors involve a hetero-ring system with a fused benzamido group that mimics nicotinamide from NAD^+^ by burial deep within the nicotinamide active-site crevice. Ring stacking occurs between Tyr493 and 504 (cholix toxin) which provides a nonpolar cradle for aromatic rings systems found in these inhibitor compounds. Additionally, a critical H-bond forms in the toxin active-site between the conserved Gly461 (Y-H-G motif) and the inhibitor imide group (Figure 6, bottom panel). The detailed mode of interactions for each inhibitor with toxin active-site residues are shown as two-dimensional drawings (Figure 7).

### 3.2. Vibrio cholerae Cholix Toxin

Cholix toxin was discovered in strains of *V. cholera* and identified through a bioinformatic strategy in 2008 [63]. It is the third toxin in the DT-group of mARTs and, like ExoA and DT, it modifies elongation factor 2 at the diphthamide residue which blocks protein synthesis in the host cell [30,64]. It is found in nearly 50% of non-O1/O139 *V. cholerae* strains and has been proposed as a major toxin and virulence factor associated with non-pandemic strains [65]. Cholix maintains *V. cholerae* fitness by promoting symbiotic interactions with eukaryotic aquatic inhabitants. Cholix may also attenuate virulence in the human gut associated with gastrointestinal disorders [66]. Diarrhea outbreaks in Denmark, Kenya, and Peru demonstrated a role for cholix toxin in non-O1/O139 *V. cholerae* isolates [67,68,69]. It was shown that two toxin types, chxA I and chxA II, were responsible for severe liver damage in mice [66]. Subsequently, Prohibitin 1 was identified as a target for cholix during *V. cholerae*-induced mitochondrial dysfunction and cytoskeletal disruption involving apoptosis in infected hepatocytes [70]. Notably, the frequency of the *chxA* gene in *V. cholerae* strains was mostly independent of the presence of other major virulence genes.

**Figure 8:**
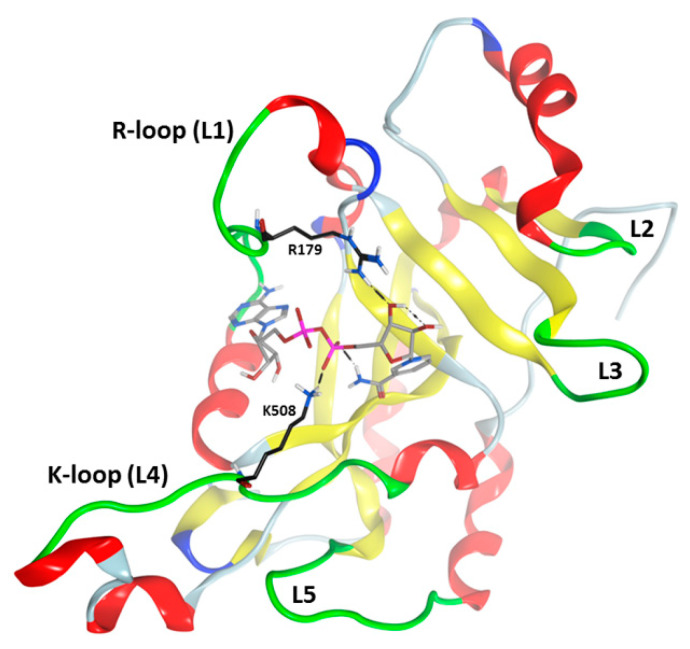
Cholix toxin active-site loops. Cholix-NAD^+^ complex ribbon revealing loops within the active-site (green). The L1–L4 loops (substrate binding) and the L4–L5 loops (target recognition) are also shown. The loop nomenclature is taken from the ExoA loop definition; for cholix toxin, the active-site loops include L1: Arg^471^-Thr^483^; L2: Thr^544^- Pro^547^; L3: Glu^574^-Glu^579^; L4: Gly^503^-Gly^512^; and L5: Gly^601^-Asp^610^. Arg^479^ and Lys^508^ C-atoms are shown in black and L1 and L4 loops are designated as R- and K-loops, respectively in cholix. The bound NAD^+^ substrate is depicted in grey C-atoms.

Cholix is a three-domain protein with a receptor-binding domain that recognizes the LRP-receptor (low-density lipoprotein receptor-related protein), a module for crossing the host cytoplasmic membrane, and a catalytic domain [71,72]. Cholix is toxic to yeast as a test-model eukaryote and catalytic signature variants demonstrated that cell death is elicited by its ADP-ribosyltransferase activity [30]. Disulfide bond reduction activates cholix coupled with furin-like protease cleavage of an arginine-rich loop. The newly formed catalytic fragment enters the host cytoplasm and ceases ribosomal protein synthesis [30]. A mobile active site loop (R-loop, L1: Arg471-Thr483) forms a solvent cover to prevent water access to the reaction center and to stabilize the reaction transition state species [64] (Figure 8). The K-loop (L4) (Gly503-Gly512) along with L5 (Gly601-Asp610) are responsible for target protein (eEF-2) recognition and binding and hence controls both GH and ADP-ribosyltransferase activities [71].

Cholix produces highly diffracting protein crystals in complex with the PARP inhibitor, PJ34, whereas the apo-cholix does not produce useful crystals [30]. Cholix-specific inhibitors were developed from two small chemical libraries. The first library is known PARP inhibitors and the second was built from a virtual screen of the cholix-PJ34 structure (PDB:2Q6M). The hallmark features for cholix active-site inhibitors center around a benzamido moiety within a hetero-ring structure that mirrors the NAD^+^ substrate nicotinamide ring [62,73]. Four cholix inhibitor scaffolds have been identified, (1) a water-soluble phenanthridinone platform (e.g., PJ34), (2) a nonpolar naphthalimide backbone (e.g., NAP), (3) PARP-like heterocyclic drugs (e.g., P6), and (4) a virtual screen-based library (e.g., V30) (Figure 6). An important feature of cholix inhibitors is the H-bond that forms with the inhibitor cyclic amide moiety and the conserved Gly residue backbone (Y-H-G motif) within DT-group toxins active site [39,40]. Remarkably, DT-group toxins, such as cholix, ExoA, and DT, bind the NAD^+^ substrate in an unusual conformation compared to dehydrogenases/NAD^+^-binding enzymes meaning that these inhibitor compounds show diminished toxicity in the host eukaryote [39,74]. PJ34 does not protect cells from cholix toxin largely due to its high aqueous solubility; however, NAP, V30, and P6-related compounds exhibit strong efficacy (EC_50_ values range from 170 nM to 4.5 μM) in target cell assays [39] (Table 2).

**Figure 9:**
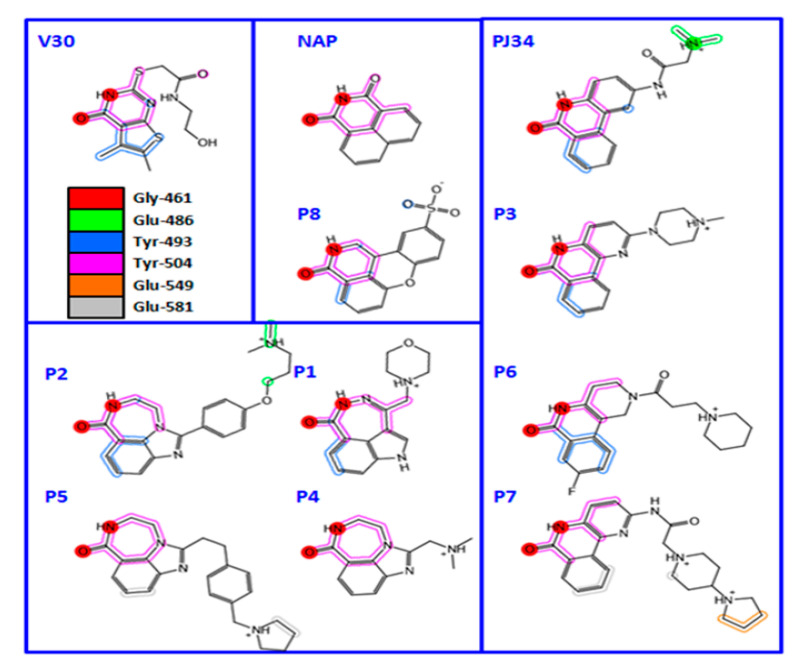
The topology drawings of V30, PJ34, and P-series inhibitors bound within the cholix active site. The pocket residues interacting with the inhibitors are shown as colors and their identities are indicated in the legend. The two red circles show the common interaction with active-site side chains.

The mode of complex formation between cholix and active-site inhibitors was evaluated based on several cholix-inhibitor structures (Figure 9) [75]. In all but V30, the inhibitors possess three pharmacophore traits of nicotinamide of the NAD^+^ substrate that include (1) H-bonds with an amide group, (2) an aromatic core formed by a pyridinium ring, and (3) a hydrophobic core at the N-site. A quantitative structure–activity relationship (3D-QSAR) analysis revealed that inhibitor binding energy arises from specific interactions of Arg479 (L1 loop), Lys508 (L4 loop), Glu481, and Gly461, in addition to others. Inhibitor V30 is unique since it lacks a fused benzamide ring as part of the core scaffold and the cholix-V30 complex (PDB:3NY6) features (1) a rotated ring system compared with nicotinamide (NAD^+^), (2) two out-of-plane methyl groups in the N-subpocket, and (3) interaction between a sulfur atom at the “tail” and the Gly461 residue via a “sigma-hole” type bond [73].

### 3.3. Vibrio splendidus Vis Toxin

*Vibrio splendidus* is a bioluminescent Gram-negative flagella bacterium in the *Vibrionaceae* family that causes diseases in mollusks responsible for vibriosis in marine life that can spread to humans upon ingestion of contaminated organisms [76]. *V. splendidus* strain 12B01 has negatively impacted the shellfish industry via infection of the Pacific oyster (*Crassostrea gigas*), leading to substantial financial losses. In silico work revealed Vis toxin as a putative virulence factor that is secreted as a 28-kDa single-domain toxin of *V. splendidus* [28]. It was characterized as a new mART enzyme with both GH and ADP-ribosyltransferase activities and its X-ray structure was determined in 2015 [32].

**Figure 10:**
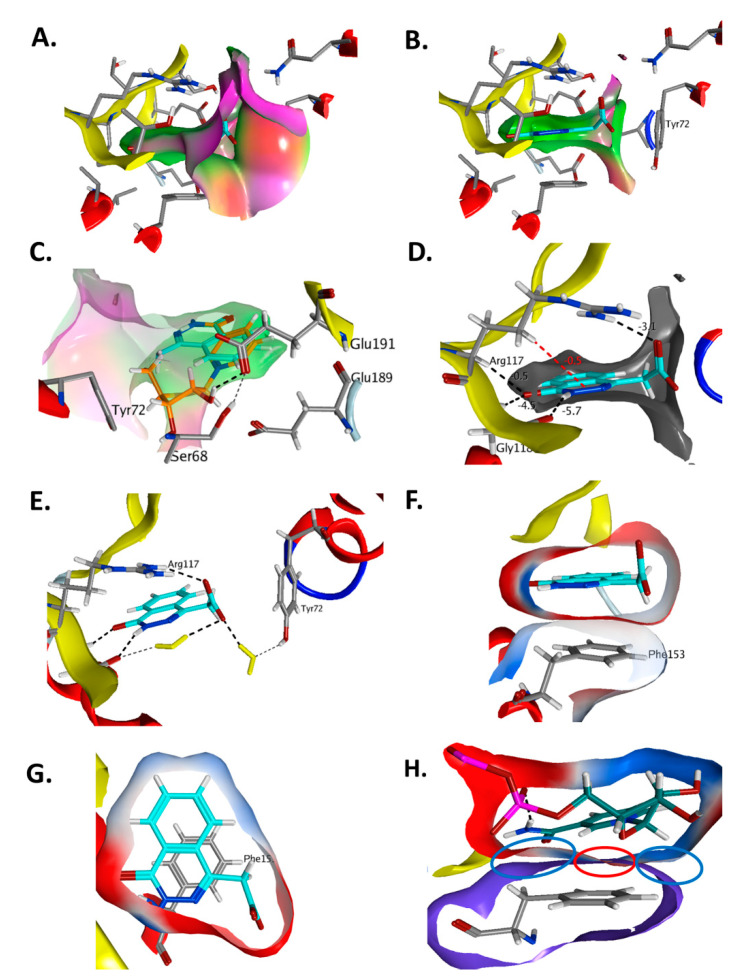
Vis interactions with NAD^+^ substrate and M6 inhibitor. H-bonds and interacting surfaces between pocket Vis residues and M6 are shown (in cyan C-atoms, panels A–G) and NAD^+^ (panel H). (**A**) Pocket residues are shown that define the van der Waals surface around M6. (**B**) Slice of the surface in (**A**), showing the N-subpocket with the M6 ring-system; the acetate carboxylate protrudes from the pocket. An empty sub-cavity is observed near Tyr^172^. (**C**) Superposition of three main pharmacophoric properties of the Vis receptor. NAD^+^ active pose (C-atoms in orange) is shown within the Vis-NAD^+^ complex, superposed onto the Vis-M6 pocket. There is a clear match between the amide group of NADs^+^ and the carbonyl and secondary amine of M6, with co-localization of the M6 benzene ring with the pyridinium ring of NAD^+^. The location of the NAD^+^ N-ribose occupies the void volume described in (**B**) by interacting with catalytic residues at the E-X-E motif (Glu^189^ and Glu^191^). (**D**) H-bond network between M6 and Arg^117^ and Gly^118^ of Vis. Arg^117^ is an H-donor by three different H-bond types (including an H-Pi type, red dashed line), binding the ligand at three points. In contrast, Gly^118^ participates in two conserved reciprocal H-bonds with the functional group at the core of M6. The numbers shown are the strength of the H-bond interaction in kcal/mol. (**E**) Anchoring of the M6 tail via H-bonds with Arg^177^ and two water-mediated bridges with Gly^118^ (left) and Tyr^72^ (right). (**F**) Lateral view of Phe^153^ and M6 with their molecular surfaces colored by electrostatic potential (blue positive, red negative). This slice shows that the planar moieties make favorable van der Waals interactions through contact with both surfaces. (**G**) Upper view of Phe^153^ and M6 by 90^°^ rotation of the perspective in panel (**F**). Thus, dihydropyridazine ring of M6 is mainly responsible for the stacking interactions with Phe^153^. (**H**) Phe^153^ and NAD^+^ are shown in the Vis-NAD^+^ complex. The substrate and side-chain moieties make weak van der Waals contacts because their molecular surfaces either clash (red oval) or are not in contact (blue ovals). The figure was taken from reference [32].

**Figure 11:**
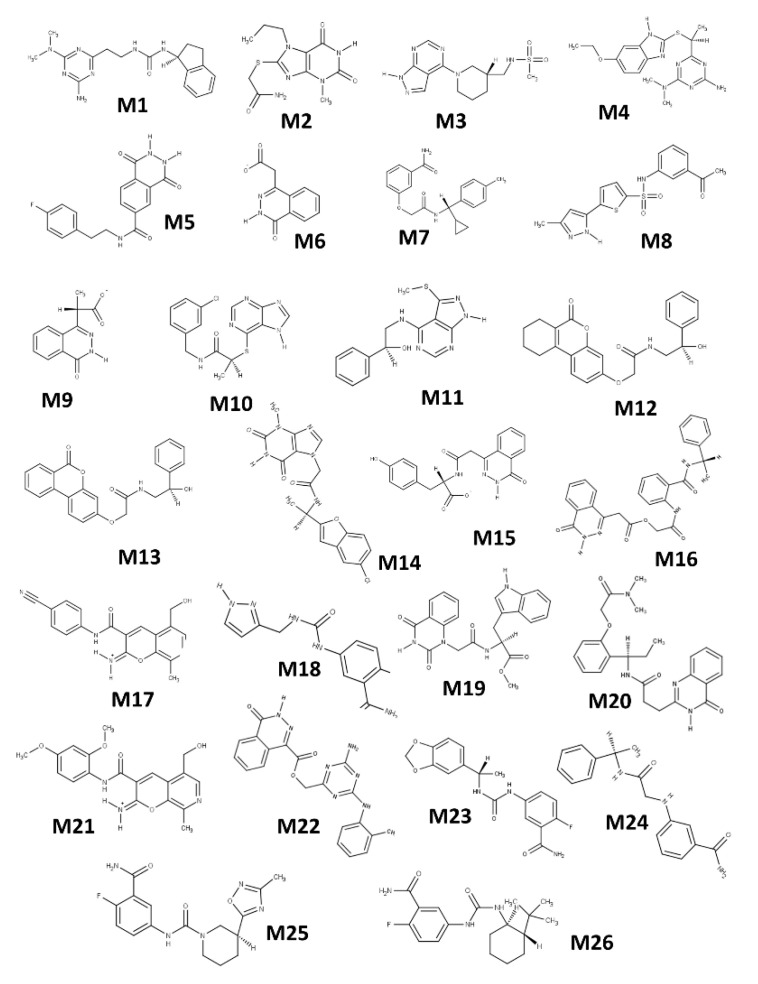
M-series inhibitors tested against Vis ADP-ribosyltransferase activity. The highest-scoring compounds from a virtual screen against iota toxin in complex with NADH (PDB code: 1GIQ) (designated the M-series) were chosen for testing as inhibitors against Vis ADP-ribosyltransferase enzyme activity. **M1**: 1-[2-[4-amino-6-(dimethylamino)-1,3,5-triazin-2-.yl]ethyl}-3-[(1S)-2,3-dihydro-1H-inden-1-yl]urea); **M2**: 2-[(3-methyl-2,6-dioxo-7-propyl-2,3,6,7-tetrahydro-1H-purin-8-yl)sulfanyl]acetamide; **M3**: N-[[(3R)-1-{1H-pyrazolo[3,4-d]pyrimidin-4-yl]piperidin-3-yl]methyl]methanesulfonamide; **M4**: 6-[(1S)-1-[(6-ethoxy-1H-1,3-benzodiazol-2-yl)sulfanyl]ethyl]-N2,N2-dimethyl-1,3,5-triazine-2,4-diamine; **M5**: N-[2-(4-fluorophenyl)ethyl]-1,4-dioxo-1,2,3,4-tetrahydrophthalazine-6-carboxamide; **M6**: 2-(4-oxo-3,4-dihydrophthalazin-1-yl)acetate; **M7**: 3-([[(S)-cyclopropyl(4-methylphenyl)methyl]carbamoyl]methoxy)benzamide; **M8**: N-(3-acetylphenyl)-5-(3-methyl-1H-pyrazol-5-yl)thiophene-2-sulfonamide; **M9**: (2S)-2-(4-oxo-3,4-dihydrophthalazin-1-yl)propanoate; **M10**: (2S)-N-[(3-chlorophenyl)methyl]-2-(7H-purin-6-ylsulfanyl)propanamide; **M11**: (1S)-2-[[3-(methylsulfanyl)-1H-pyrazolo[3,4-d]pyrimidin-4-yl]amino]-1-phenylethan-1-ol; **M12**: N-[(2R)-2-hydroxy-2-phenylethyl]-2-([6-oxo-6H,7H,8H,9H,10H-cyclohexa[c]chromen-3-yl]oxy)acetamide; **M13**: N-[(2R)-2-hydroxy-2-phenylethyl]-2-([6-oxo-6H-benzo[c]chromen-3-yl]oxy)acetamide; **M14**: N-[(1R)-1-(5-chloro-1-benzofuran-2-yl)ethyl]-2-(3-methyl-2,6-dioxo-2,3,6,7-tetrahydro-1H-purin-7-yl)acetamide; **M15**: (2S)-3-(4-hydroxyphenyl)-2-[2-(4-oxo-3,4-dihydrophthalazin-1-yl)acetamido]propanoate; **M16**: [(2-[[(1S)-1-phenylethyl]carbamoyl]phenyl)carbamoyl]methyl 2-(4-oxo-3,4-dihydrophthalazin-1-yl)acetate; **M17**: 3-[(4-cyanophenyl) carbamoyl]-5-(hydroxymethyl)-8-methyl-2H-pyrano [2,3-c]pyridin-2-iminium; **M18**: 2-fluoro-5-[[(1H-pyrazol-3-ylmethyl)carbamoyl]amino]benzamide; **M19**: methyl (2S)-2-[2-(2,4-dioxo-1,2,3,4-tetrahydroquinazolin-1-yl)acetamido]-3-(1H-indol-3-yl)propanoate; **M20**: N-[(1R)-1-[2-[(dimethylcarbamoyl)methoxy]phenyl]propyl]-3-(4-oxo-3,4-dihydroquinazolin-2-yl)propanamide; **M21**: 3-[(2,4-dimethoxyphenyl)carbamoyl]-5-(hydroxymethyl)-8-methyl-2H-pyrano[2,3-c]pyridin-2-iminium; **M22**: [4-amino-6-[(2-methylphenyl) amino]-1,3,5-triazin-2-yl]methyl 4-oxo-3,4-dihydrophthalazine-1-carboxylate; **M23**: 5-([[(1S)-1-(2H-1,3-benzodioxol-5-yl)ethyl]carbamoyl]amino)-2-fluorobenzamide; **M24**: 3-[([[(1R)-1-phenylethyl] carbamoyl]methyl) amino]benzamide; **M25**: (3S)-N-(3-carbamoyl-4-fluorophenyl)-3-(3-methyl-1,2,4-oxadiazol-5-yl)piperidine-1-carboxam; **M26**: 5-([[(1R,2S)-2-tert-butyl cyclohexyl] carbamoyl]amino) -2-fluorobenzamide. Figure was taken from reference [32].

A virtual screen was conducted against iota toxin in complex with NADH (PDB:1GIQ) which produced a library of 294 compounds (M-series) for in vitro and cell-based testing [32]. Twenty-six compounds from the M-series were found to inhibit Vis toxin and several were characterized for inhibition constants, K_i_. Experimental testing revealed six M-series compounds that showed K_i_ values below 100 μM (M2, M3, M4, M6, M9, and M19) and the structure of Vis with the M6 inhibitor was determined at 1.50 Å (Figure 10). The M-series inhibitors in order of potency were M2 (a substituted purine), M4 (a substituted benzodiazo), and M19 (a diazinanedione coupled to an indole via a branched methyl-methylene-amide bridge) (Figure 11). Inhibitors M6, M9, and M15 are oxy-dihydrophthalazine compounds with acetate, propanoate, and phenyl amido propanoate side-chains, respectively (Figure 11). M3 features a pyrazolo-pyrimidine scaffold N-linked to a piperidine ring that holds a methane sulfonamide moiety. M18 gave a K_i_ = 134 µM and it features a fluorobenzamide ring coupled to a pyrazolidine ring with an amide methylamino linker. Compounds M10, M24, and M25 showed inhibitory activity but were weaker inhibitors (K_i_ ≈ 230, 350, and 630 µM, respectively).

M6 is a heterocyclic with a benzene ring fused to dihydropyridazinone harboring an acetate substituent. The Vis-M6 X-ray structure revealed several Vis side-chains that pack around the M6 ligand and exclude water from the active site. The M6 benzyl moiety overlaps the nicotinamide ring when superposed with the NAD^+^ substrate. The methylene groups of Glu189 and Glu191 side-chains contact the M6 benzene ring hydrogens. Gly118 and Arg177 coordinate the M6 inhibitor with strong H-bonds and electrostatic interactions with the acetate substituent. Overall, there are 15 heavy atoms from M6 that interact with 16 Vis residues [32].

### 3.4. Streptomyces scabies Scabin Toxin

*Streptomyces scabies* is an actinomycete, Gram-negative bacterium that residues in the soil throughout the world. It is a global pathogen of tuberous and root crops including potatoes, yams, carrots, turnips, etc. causing corky lesions in the epidermal layer that negatively impacts the market value of vegetable produce [77]. The *S. scabies* infection begins when germinating spores invade plant tissue through lesions and lenticels (cracks) in the vegetable [78] and produces a key phytotoxin that contributes to disease symptom development [79]. Only rapidly expanding areas of the tuber will be affected, allowing the scabs or lesions to grow as the plant tuber expands. Once the plant tuber has fully grown, the lesion will cease growing. Infection of roots is usually not as evident as the corky lesions observed on the surface of the vegetable; root stunting, browning, and seedling death may occur. Recently, biocontrol agents have been shown to suppress *S. scabies* growth and help curb the common scab disease [80,81].

A novel in silico strategy identified Scabin as a putative virulence factor of *S. scabies* strain 87.22 [29]. Scabin is secreted in the extracellular milieu and uses the Tat secretion pathway and its signal peptide to exit the producing bacterium to gain access to the host plant [82]. Scabin harbors the common catalytic features of the CT-group mART toxins, including the NAD^+^-binding S-T-T motif, and the catalytic Arg and Q-X-E regions. Scabin has been grouped with the Pierisin family of mART toxins since it shares large, conserved regions in the catalytic core with Pierisins [24]. Scabin has been well characterized with a full kinetic analysis of its GH and ADP-ribosyltransferase functions. It has been shown to modify small nucleosides/nucleotides, RNA, DNA, and genomic DNA as substrates [24,34]. An HDX study of Scabin in complex with ss-DNA revealed some important insights into its DNA-binding footprint within the active-site cleft of the enzyme [83]. Several crystal structures of the enzyme with and without substrate analogues and inhibitors have been solved and a large library of catalytic variants have been produced and kinetically analyzed [24,34,83].

**Figure 12:**
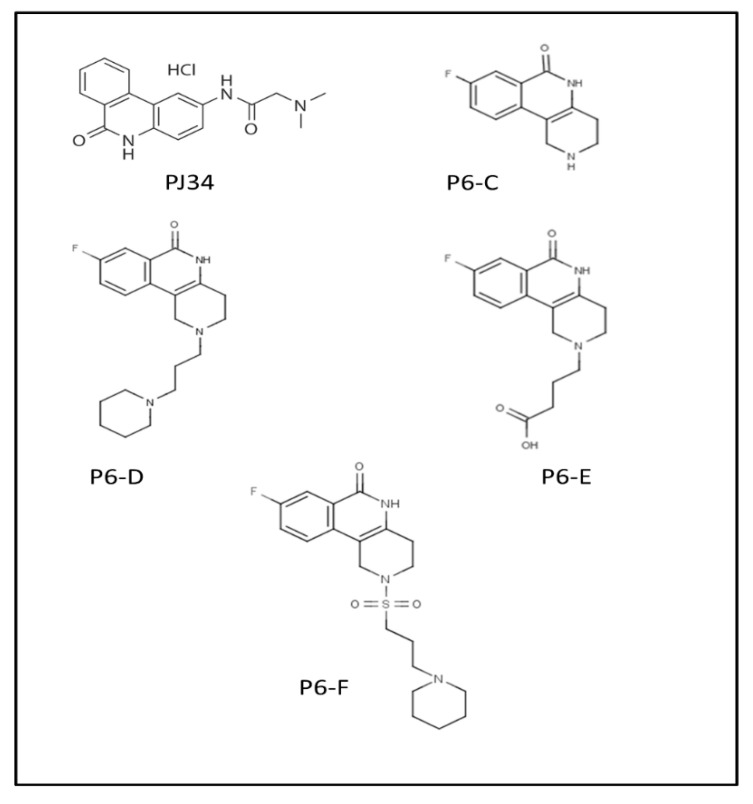
P-series inhibitors effective against Scabin GH activity. The chemical structures of the following Scabin inhibitors are shown: PJ34, 2-[[3-(dimethylamino)-2-oxopropyl]amino]-5,6-dihydrophenanthridin-6-one; P6-C, 8-fluoro-1H,2H,3H,4H,5H,6H-benzo[c]1,6-naphthyridin-6-one; P6-D, 8-fluoro-2-[3-(piperidin-1-yl)propyl]-1H,2H,3H,4H,5H,6H-benzo[c]1,6-naphthyridin-6-one; P6-E, 4-[8-fluoro-6-oxo-1H,2H,3H,4H,5H,6H-benzo[c]1,6-naphthyridin-2-yl]butanoic acid; P6-F, 8-fluoro-2-[3-(piperidin-1-yl)propanesulfonyl]-1H,2H,3H,4H,5H,6H-benzo[c]1,6-naphthyridin-6-one. Figure was taken from reference [24].

A series of inhibitors were tested against Scabin GH activity based on a library of compounds designed to act as competitive inhibitors against the NAD^+^ substrate [24]. Five compounds were found to inhibit Scabin enzyme activity including PJ34, P6-C, P6-D, P6-E, and P6-F. Kinetic analysis showed that the mechanism of inhibition was competitive with the NAD^+^ substrate (Table 3). Notably, these compounds show greater affinity for Scabin than the NAD^+^ substrate, with K_i_ values ranging from 3–50 μM. The PARP drug, PJ34, was the most promising inhibitor (K_i_ near 3 μM). The P-series compounds also showed good efficacy against Scabin with P6-F showing the highest affinity (K_i_ = 7 μM) and P6-E slightly weaker affinity (K_i_ = 24 μM). The nonpolar character of the terminal piperidine “tail” group in P6-D and P6-F increases inhibitor affinity compared with the naked P6-C benzo-napththyridinone ring system (Table 3; Figure 12) [24]. Furthermore, P6-E possesses an anionic terminal carboxylate group, and this slightly weakens binding affinity with Scabin.

**Figure 13:**
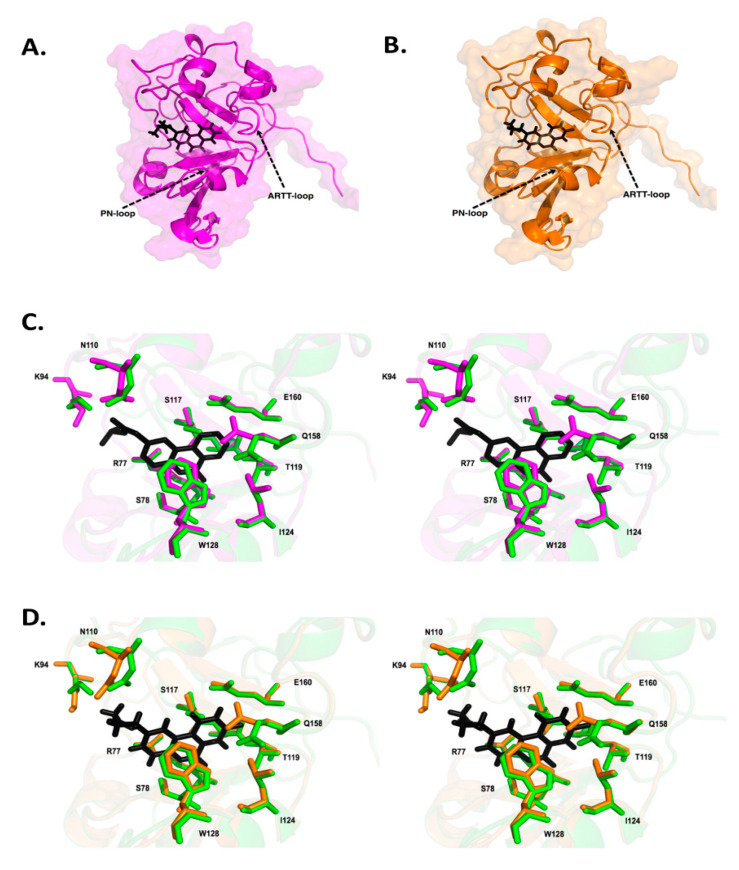
Scabin inhibitor co-crystal structures (**A**) The structure of the Scabin-PJ34 complex is shown as a ribbon diagram. PJ34 is shown in stick format (colored black). (**B**) The Scabin-P6-E complex is shown in ribbon format. P6-E is represented in stick format (black). (**C**) A stereo view of the Scabin-PJ34 active-site (magenta) and Scabin-apo structure (green). PJ34 is shown in stick format (colored black). The differences in structure among important catalytic residues (Arg^77^ Ser^78^, Lys^94^, Asn^110^, Ser^117^, Thr^119^, Leu^124^, Try^128^, Gln^158^, Glu^160^) are highlighted. (**D**) Stereo view of the Scabin-P6-E complex structure (magenta) and Scabin-apo structure (green). P6-E is represented in stick format (black). Structural differences are highlighted among important catalytic residues (Arg^77^ Ser^78^, Lys^94^, Asn^110^, Ser^117^, Thr^119^, Leu^124^, Try^128^, Gln^158^, Glu^160^). Figure was taken from reference [24].

The crystal structures of Scabin in complex with PJ34 and P6-C compounds provided further insight into the inhibitor features necessary for forming tight complexes with Scabin [24]. The catalytic residues found in the ARTT loop responsible for substrate binding orient towards these inhibitors with a 2.5 Å shift of Gln158. The PJ34 inhibitor is nestled within the Scabin active-site through nonpolar interactions coupled with H-bonds (Figure 13). Two H-bonds form with the Ser78 main-chain nitrogen and oxygen atoms. Another key H-bond forms between Asn110 and the PJ34 tertiary amine R-group. Gln158 in the Q-X-E motif shifts 2.5 Å upon P6-E binding in the Scabin active-site. The P6-E inhibitor is cradled in the active site with nonpolar contacts and 2 H-bonds. One H-bond forms between Arg77 guanindine side-chain and the carboxyl group of P6-E (3.4 Å long). Another H-bond forms between the P6-E oxygen and the Ser78 main-chain nitrogen (2.8 Å long). Overall, the Scabin-P6-E complex resembles that of Scabin-PJ34 in structural orientation and position. In summary, the changes within the Scabin structure upon inhibitor binding are modest in nature with the largest change associated with the re-orientation of the ARTT loop resulting in a 2.5 Å shift in Gln158. Apparently, inhibitor/ligand binding within the N-subsite of Scabin triggers a change in Gln158 side-chain conformation towards an inward-facing disposition. Nonpolar and uncharged inhibitor “tails” are preferred to negatively charged (carboxylate) or positively charged (amino) moieties.

NADH is a strong, competitive inhibitor against the NAD^+^ substrate with Scabin (K_i_ = 1.5 μM) and the stronger binding of the reduced dinucleotide was attributed to increased van der Waals interactions with two H-atoms (out-of-plane) at the pyridine C4 position in nicotinamide [34]. This is coupled with a more favorable electrostatic complex resulting from the loss of charge of the pyridine N1 atom in NADH compared with NAD^+^. Several key side-chains of Scabin pocket residues are shifted upon NADH binding (RMSD = 0.94 Å, 24 residues); notable residues include Arg81, Lys94, Asn110, Trp128, and Gln158 (1.8 Å for the atoms in these five residues). The Trp128 side-chain rotates 180° with its indole nitrogen shifting 4 Å. A network of H-bonds coupled with steric contacts occurs between NADH and Scabin, including two reciprocal H-bonds with Ser78. The side-chains of Ser117, Thr118, T119 (STS motif), Trp 128 (PN loop), and Leu124 form a planar nonpolar pocket for the NADH nicotinamide ring. The catalytic Q-X-E motif (Q158-X-E160) at β5 contacts the N-ribose with Arg77 (conserved) and Asn110 contacts the PO_2_-O-PO_2_ linker while Lys94 forms a stable salt bridge. The adenine ring of NADH forms a planar stack with Ly94 and Arg81 with an H-bond formed by Ser80 and the A-ribose. Interestingly, there are 10 water molecules that are displaced when Scabin binds NADH. HOH39 bridges NADH helping to stabilize its binding pose. Furthermore, Asn110 bridges the A-phosphate of NADH through displacement of HOH181 and is linked to Ser117 by the rotation of HOH41.

### 3.5. Bacillus cereus Certhrax Toxin

**Figure 14:**
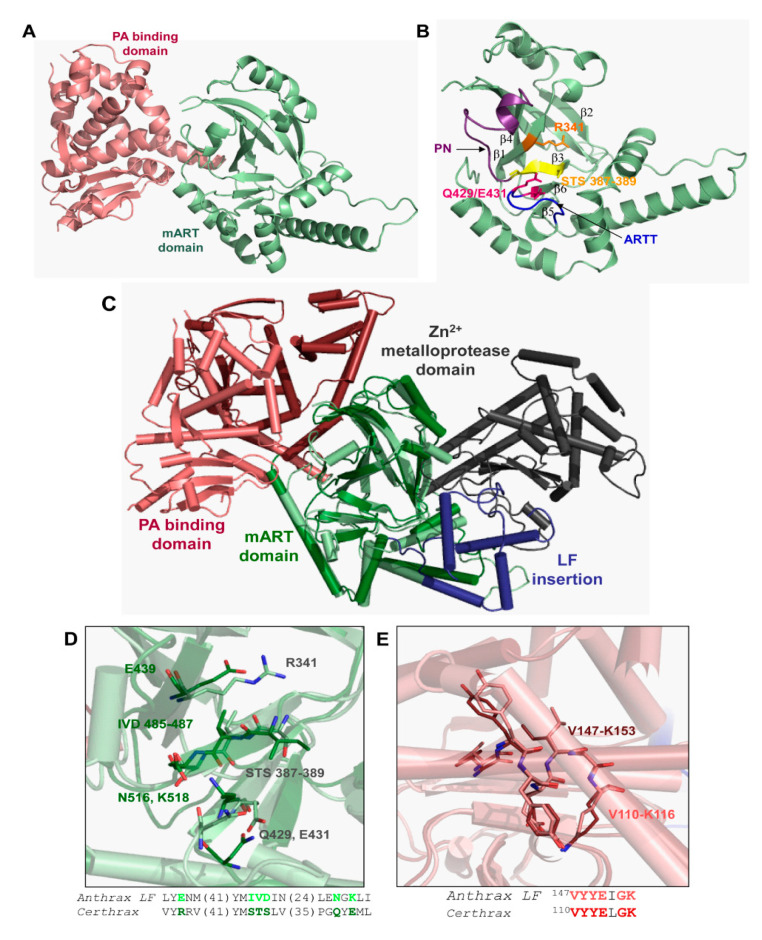
Certhrax structure. (**A**) Certhrax crystal structure with no bound ligand bound. The protective antigen (PA)-binding domain is shown in pink; the mART domain is illustrated in green. (**B**) Certhrax catalytic domain. Important catalytic residues are shown in orange stick representation (R341), magenta (Q429, E431), and yellow (STS motif 387–389). The PN loop is shown in purple, and the ARTT loop is highlighted in blue. (**C**) Superposition of Certhrax (pale colors) with anthrax LF (bold colors) and they are aligned with respect to the mART domains. The domains colored as in Figure 1B with the additional anthrax LF insertion domain and zinc metalloprotease domain in blue and grey, respectively. (**D**) Important Certhrax active site residues are shown in light green and anthrax LF aligned residues are depicted in dark green. Residues not shown in the amino acid sequence are indicated as numbers in parentheses. (**E**) Residues important for PA binding are structurally aligned in Certhrax (pink) and anthrax LF (red). Residues shown in stick representation are highlighted and are shown as text sequence beneath. Figure was taken from reference [31].

In silico data mining of bacterial genomes uncovered a C2-like mART toxin, Certhrax, from a pathogenic strain (G4291) of *B. cereus* [31]. Certhrax was characterized kinetically, its structure was determined, and inhibitors were identified [31], followed by target substrate identification (vinculin) and cell biology studies [84,85]. The Certhrax structure revealed high structural similarity with two domains of anthrax lethal factor but Certhrax lacks the metalloprotease domain (Figure 14). The Certhrax structure includes a protective antigen (PA)-binding domain at the N-terminus and an mART domain at the C-terminus. Certhrax was shown to be highly toxic to yeast cells and to RAW264.7 murine macrophages in culture [31]. It was shown to modify vinculin with ADP-ribose which leads to the disruption of focal adhesion complexes and a redistribution of vinculin to the cytoplasm. This provides a means for the *B. cereus* pathogen to invade host cells and proliferate [84].

Based on a virtual screen followed by a cell-based screening, twelve inhibitors were identified that showed efficacy against Certhrax toxin including the P6-series inhibitors (P1, P3, P6) and some P6 derivatives (P6C, P6D, P6F, P6G), PJ34, PJ197A, V23, V30, and suramin (Table 4). These inhibitors are competitive inhibitors against the NAD^+^ substrate and P6 (8-fluoro-2-[3-(piperidin-1-yl)propanoyl]-1H,3H,4H,5H-benzo[c]1,6-naphthyridin-6-one) was the most potent inhibitor with K_i_ and K_D_ values of 1.7 and 1.8 μM, respectively [31]. P6 was further tested in mouse macrophages treated with Certhrax. P6 showed excellent ability to protect macrophages from Certhrax intoxication (EC_50_ = 5.7 μM) in the presence of 500 times the lethal dose of the toxin.

**Figure 15:**
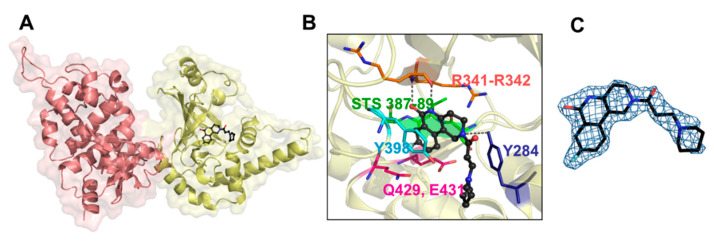
Certhrax structure with bound inhibitors. (**A**) Certhrax (PA-binding domain, pink; mART domain, yellow) in complex with P6 inhibitor (black). (**B**) P6 interactions with Certhrax active-site residues. Active-site residues are shown in stick representation, and P6 is shown in ball-and-stick (black). Arg342 (orange) and Tyr284 (blue) form H-bonds with the inhibitor (dashed lines), while Tyr398 (cyan) has aromatic interactions. Additional active site residues are depicted in pink (Q-x-E motif) and green (STS motif). (**C**) P6 electron density is shown when bound to Certhrax. Simulated annealing omit map around the inhibitor is shown in blue (contoured at 1σ). Figure was taken from reference [31].

The structure of Certhrax with P6 is shown in Figure 15. The P6 phenanthridinone ring is buried in the Certhrax substrate pocket and is embraced by both the PN and ARTT loops. P6 is tethered by H-bonds with Arg342 on β1 strand, Tyr284 on the α3 helix, and π-stacking interaction with Tyr398 on the PN loop [31]. The PJ34 ligand has its 6-oxo-5,6-dihydrophenanthridine moiety embedded within the Certhrax substrate pocket with H-bonds to Arg342 and an ordered water molecule while the N, N-dimethyl acetamide tail is quite disordered. Again, the common theme for mART active-site inhibitors was observed for these two protein-ligand complexes; the root-mean-square deviation (RMSD) of the apo-Certhrax structure with the P6- and PJ34-bound structures was 0.59 Å and 0.46 Å, respectively. The high similarity of Certhrax-inhibitor structures is partly due to the movement of the PN loop (12 Å at its furthest point) that includes Tyr398 which forms aromatic interactions with both inhibitors. Notably, unlike other mART enzymes, the ARTT loop in Certhrax does not shift significantly upon inhibitor binding [31].

### 3.6. Paenibacillus larvae C3larvin and Plx2A

American Foulbrood (AFB) is a problematic brood disease for *Apis mellifera* beekeepers world-wide [86] and is responsible for major economic damage in global agriculture associated with dwindling pollinator populations [87]. *Paenibacillus larvae* is the causative agent of AFB with two genotypes, ERIC I and ERIC II, participating in contemporary outbreaks globally. Two mART toxins have been shown as important *P. larvae* ERIC I virulence factors [88] with Plx2A recently characterized and its crystal structure solved [37]. An ERIC II putative virulence factor, C3larvin, was also characterized, but was shown to be non-functional in the honeybee pathogen [33,37]. However, in vitro, C3larvin was shown to modify RhoA as a substrate and to be toxic to a yeast host when expressed cytoplasmically [33].

**Figure 16:**
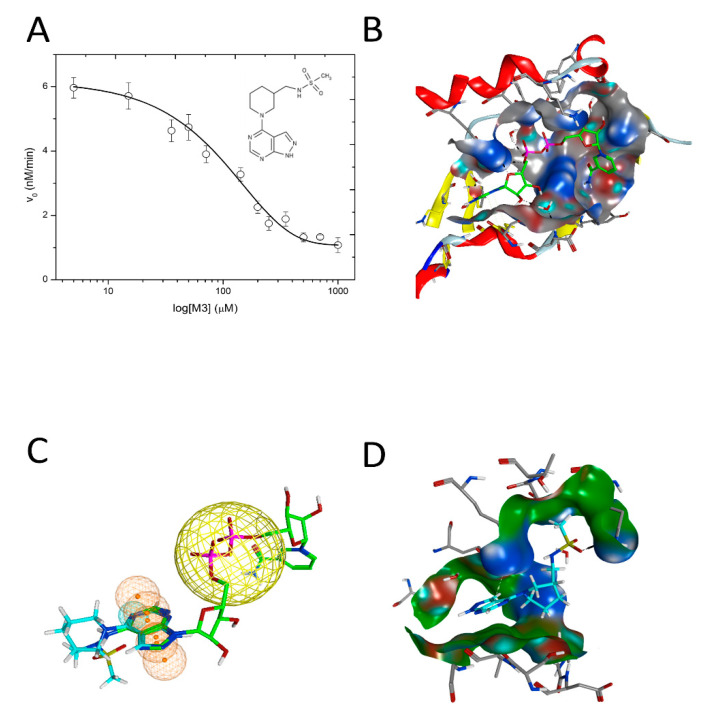
C3larvin GH activity inhibited by M3. (**A**) C3larvin dose–response with M3 inhibitor. M3 inhibitor caused the loss of GH activity as described under Experimental Procedures and the IC_50_ value was calculated from the data. Error bars, S.D. from at least three experiments. Inset to (**A**): Structure of the M3 inhibitor, N-[(1-[1H-pyrazolo[3,4-d]pyrimidin-4-yl]piperidin-3-yl)methyl]methanesulfonamide. (**B**) C3larvin pocket definition (gray surface) based on the NAD^+^ active conformation (green C-atoms). (**C**) C3larvin pharmacophore model. C3larvin with modeled NAD^+^ and with M3 (cyan C-atoms) superposed (manually) to the adenine ring-system, to depict the common features. The pharmacophore definition based on the NAD^+^ adenine moiety is shown as orange spheres/mesh and the anion-center feature is depicted as a large yellow sphere. (**D**) Docked poses of M3 (cyan C-atoms), with only the features at the adenine moiety and an induced fit (flexible) receptor. The M3 ring-system is rotated relative to the previous slide. Figure was taken from reference [33].

A small molecule inhibitor of C3larvin, M3 (N-[(1-[1H-pyrazolo[3,4-d]pyrimidin-4-yl]piperidin-3-yl)methyl]methanesulfonamide), was identified from a virtual library screen [32] and was shown to inhibit the enzyme with a K_i_ = 11 μM [33] (Figure 16). It represents the first known inhibitor of mART C3 toxin subgroup. M3 chemically differs from previous mART inhibitors since it consists of an adenine ring linked to a piperidine substituted with a sulfated amine. Since the C3larvin-M3 complex did not produce diffraction quality crystals, a molecular mechanics/molecular dynamics approach was used to define the inhibitor-binding pocket and pharmacophore model (Figure 16) [89]. It was suggested that M3 competes with the adenine ring of the NAD^+^ substrate but verification must await a high-resolution structure of the C3larvin-M3 complex. Preliminary work indicates that flavonoids are excellent natural product inhibitors of the *P. larvae* toxins such as C3larvin, Plx2A, and an ERIC III virulence factor, C3larvinA. Current work is focused on the structural and functional characterization of these plant-based compounds as inhibitors of C3 subgroup mART toxins.

## 4. Conclusions

The therapeutics arising from anti-virulence strategies can be expected to have broad application in combating plant, animal, and human diseases [90]. Indeed, virulence factors identified and characterized from pathogenic bacteria provide important new drug targets in the fight against antibiotic-resistant microbes. This anti-virulence strategy against mART toxins features the ability to iteratively screen, test, and optimize for inhibitors that protect yeast cells (model system), animal (including human), and plant cells from the cytotoxic effects of these toxins, thus, providing an important prophylactic approach in the quest to disarm disease-causing bacteria [91,92].

In conclusion, several libraries of mART inhibitors against both DT- and CT-group toxins have been identified from a combination of PARP-based inhibitors, virtual screening, and rational inhibitor design. Future work will undoubtedly entail refinement of the potency of these lead compounds through a combination of Structure-Activity-Relationships (SARs) and Quantitative-Structure-Activity-Relationships (QSARs). An attractive approach is to use plants as the source for the discovery of natural products to target bacterial virulence factors [93,94]. Recently, a family of plant metabolites known as flavonoids have been identified as strong inhibitors of C3-like mART toxins and will provide the basis for the development of potent and less-toxic anti-virulence agents to treat bacterial disease.

## Figures and Tables

**Table 1:** ExoA inhibitors.

Compound	Inhibitor Name	Structure	IC_50_ (μM)
PJ-34	N-(6-oxo-5,6-dihydro-phenanthridin-2-yl)-N,N-dimethylacetamide	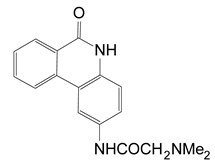	0.296 ± 0.080
D	3-(morpholin-4-ylmethyl)-1,5-dihydro-6*H*-[1,2]diazepino[4,5,6-*cd*]indol-6-one	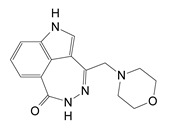	0.617 ± 0.023
F	1-[4-(3-dimethylamino-propoxy)-phenyl]-8,9-dihydro-*7H*-2,7,9a-triaza-benzo[*cd*]azulen-6-one HCl	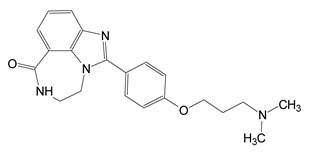	0.495 ± 0.090
G	2-(4-methylpiperazin-1-yl)-5*H*-benzo[*c*][1,5]naphthyridin-6-one MsOH	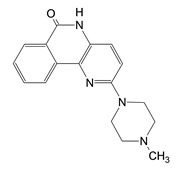	1.965 ± 0.032
H	1-dimethylaminomethyl-8,9-dihydro*-7H*-2,7,9a-triaza-benzo[cd]azulen-6-one HCl	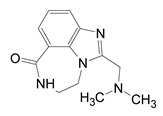	0.964 ± 0.050
I	1-[2-(4-pyrrolidin-1-ylmethyl-phenyl)-ethyl]-8,9-dihydro-*7H*-2,7,9a-triaza-benzo[*cd*]azulen-6-one hydrochloride HCl	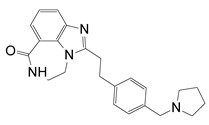	6.52 ± 0.39
L	8-fluoro-2-(3-piperidin-1-ylpropanoyl)-1,3,4,5-*2H-*tetrahydrobenzo[*c*]-1,6-naphthyridin-6-one MsOH	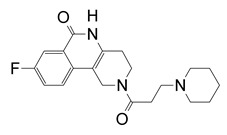	1.542 ± 0.044
M	*N*-(6-oxo-5,6-dihydrobenzo[*c*][1,5]naphthyridin-2-yl)-2-(4-pyrrolidin-1-ylpiperidin-1-yl)acetamide·HCl	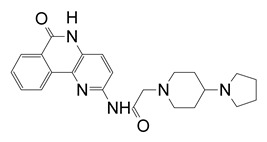	0.375
N	2-(4-isopropylpiperazin-1-yl)-5*H*-benzo[*c*][1,5]naphthyridin-6-one MsOH	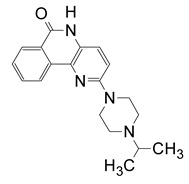	1.07
P	1,11b-dihydro-[1]benzopyrano[4,3,2-de]isoquinolin-3(2H)-one-10-sulfonic acid	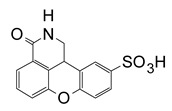	0.453

**Table 2:** Properties of inhibitors of *Pseudomonas aeruginosa* ExoA.

Compound	^a ^K_d_ (nM)	^b ^K_i_ (nM)	^c ^IC_50_ (nM)	^d ^EC_50_ (µM)
P1	10 ± 2; 650 ± 50	22 ± 4	170 ± 30	2.9 ± 0.8
P2	260 ± 10	63 ± 5	480 ± 40	^e^ n.d.
P3	1470 ± 30	90 ± 17	690 ± 130	^e^ n.d.
P4	1380 ± 30	132 ± 7	960 ± 50	12.6 ± 3.3
P5	750 ± 10	582 ± 124	4460 ± 950	16.7 ± 1.9
P6	1100 ± 20	80 ± 18	610 ± 140	3.4 ± 1.6
P7	680 ± 40	118 ± 7	908 ± 118	^e^ n.d.
P8	160 ± 30; 5210 ± 1780	136 ± 3	1040 ± 136	^e^ n.d.
V30	931 ± 74	367 ± 3	2815 ± 22	8.8 ± 0.5
NAP	950 ± 30	12 ± 1	90 ± 10	3.8 ± 0.9
PJ34	820 ± 54	37 ± 9	280 ± 70	^e^ n.d.
PJ97A	393 ± 64	610 ± 175	4674 ± 175	^e^ n.d.

^a^ The binding affinity of inhibitors to wild-type (WT) ExoA_c_ was measured by the quenching of the intrinsic Trp fluorescence caused by the binding of the ligand to the enzyme active site. ^b^ The absolute inhibition constant (K_i_) was calculated from the experimentally determined IC_50_ values according to the following relationship, K_i_ = IC_50_/(1 + ([S_NAD_]/K_M(NAD)_)^7^; see Materials and Methods for details. The [NAD^+^] was 300 µM and the K_M (NAD)_ was 45 µM. ^c^ The IC_50_ values were determined by fitting each dose–response curve to a Boltzmann Sigmoidal function in Origin 6.1. ^d^ The EC_50_ values were determined for inhibitors added to C38 cells in the presence of 650 ng/mL of ExoA in 96-well plates for 96 h. ^e^ n.d., not determined.

**Table 3:** Kinetics of Scabin inhibitors.

Inhibitor	^a ^IC_50_ (μM)	^b ^*K_i_* (μM)	^c ^pIC_50_	^d ^*K_D_* (μM)
PJ34	12 ± 1	3 ± 0.2	4.9	14 ± 0.5
P6-C	89 ± 4	19 ± 1	4.1	25 ± 1
P6-D	97 ± 7	18 ± 1	4.0	42 ± 5
P6-E	119 ± 2	24 ± 0.3	3.9	50 ± 6
P6-F	38 ± 2	7 ± 0.2	4.4	^e^ ND

^a^Each dose–response curve was fit to a Boltzmann Sigmoidal equation to determine IC_50_ values. ^b^The K_i_ (inhibition constant) was determined with the equation (K_i_ = IC_50_/(1 + ([S_NAD_]/K_M(NAD)_) using these values [Scabin], 10 µM, [εNAD^+^], 400 µM, and K_M_ (εNAD^+^) was 276 µM. ^c^The values for pIC_50_ for the inhibitors were obtained from the following: pIC_50_ = −logIC_50_. The greater the pIC_50_ value, the smaller dose is necessary for 50% inhibition of Scabin enzyme activity. ^d^Scabin binding affinity to inhibitors was determined from quenching of intrinsic fluorescence upon ligand binding to the enzyme. ^e^ND, not determined.

**Table 4:** Inhibition and binding constants of Certhrax inhibitors.

Inhibitor	*K_D_* (μM) ^a^	*IC_50_* (μM) ^b^	*K_i_* (μM) ^c^
P6	1.7 ± 0.2	6.1 ± 1.2	1.8 ± 0.4
P3	3.3 ± 0.4	7.2 ± 2.6	2.1 ± 0.8
Suramin	1.9 ± 0.7	10.6 ± 1.5	3.1 ± 0.4
P6F	3.1 ± 0.3	12.1 ± 1.1	3.6 ± 0.3
P1	1.3 ± 0.2	13.0 ± 2.7	3.9 ± 0.8
PJ97A	1.6 ± 0.6	16.8 ± 1.6	5.0 ± 0.5
PJ34	5.8 ± 2.6	32.3 ± 1.1	9.6 ± 0.3
P6D	n.d. ^d^	76.1 ± 1.2	22.5 ± 0.4
V30	n.d.	87.1 ± 1.3	25.8 ± 0.4
P6C	n.d.	121.3 ± 1.7	35.9 ± 0.5
V23	n.d.	380.2 ± 3.3	112.4 ± 1.0
P6G	n.d.	>1000	>295

^a^*K_D_* values represent the mean ± S.E. from at least three independent experiments with at least nine replicates. ^b^*IC_50_* values represent the mean ± S.E. from at least three independent experiments and at least six replicates. ^c^*Ki* values using the Cheng–Prusoff equation, *K_i_* = IC_50_/(1 + [S]/*K_m_*), where [S] is the NAD^+^ concentration and Km is for the NAD^+^ substrate. ^d^n.d., not determined. Table was taken from reference [31].

## Data Availability

Data are available upon request, please contact the contributing authors.
